# Effects of transtheoretical model-based interventions on body mass index and other health outcomes in overweight or obese populations: a systematic review and meta-analysis

**DOI:** 10.3389/fpubh.2026.1832812

**Published:** 2026-06-22

**Authors:** Jinghui Deng, Wenjun Lu, Tong Liu

**Affiliations:** 1 College of Sports and Health, Aba Teachers University, Aba, Sichuan, China; 2 College of Physical Education, Chengdu Sport University, Chengdu, Sichuan, China

**Keywords:** body mass index, health education, meta-analysis, obesity, overweight, self-efficacy, transtheoretical model

## Abstract

**Background:**

Globally, an estimated 2.2 billion adults and 430 million children and adolescents are affected by overweight or obesity. Interventions based on the transtheoretical model (TTM) have demonstrated efficacy in modifying dietary behaviors, reducing body mass index (BMI), and improving other health outcomes in overweight or obese populations. However, the magnitude of these effects varies across studies and study designs. This study aimed to systematically evaluate the impact of TTM-based interventions on BMI and other physical health indicators among individuals with overweight or obesity, with separate analyses for randomized controlled trials (RCTs) and one-group repeated measures designs (ORMDs).

**Methods:**

A systematic review was conducted following Preferred Reporting Items for Systematic Reviews and Meta-Analyses (PRISMA) guidelines, searching six databases—CNKI, PubMed, Web of Science, Cochrane Library, EBSCO, and Scopus—from their inception through March 30, 2025. Studies were eligible if they employed TTM-based interventions and reported BMI outcomes in overweight or obese populations. Hedges’ g served as the effect size measure. For ORMDs, sensitivity analyses were conducted using pre-post correlation coefficients of *r* = 0.3, 0.5, and 0.7. Random-effects meta-analyses (REML estimator) were performed separately for RCTs and ORMDs. Subgroup analyses and meta-regression were conducted for BMI outcomes to explore sources of heterogeneity. Publication bias was assessed using funnel plots and Egger’s test, and sensitivity was evaluated using leave-one-out analyses.

**Results:**

A total of 20 studies (1,736 participants) were included, comprising 9 RCTs and 11 ORMDs. Among ORMDs, TTM-based interventions significantly reduced BMI [*k* = 11, *g* = −0.467, 95% CI (−0.763, −0.171), *p* = 0.002, *I^2^* = 86.6%], body weight [*k* = 5, g = −0.323, 95% CI (−0.563, −0.084), *p* = 0.008, *I^2^* = 78.9%], and waist circumference [*k* = 2, g = −0.375, 95% CI (−0.542, −0.209), *p* < 0.001, *I^2^* = 0%], and significantly increased self-efficacy [*k* = 5, *g* = 1.195, 95% CI (0.699, 1.692), *p* < 0.001, *I^2^* = 82.6%]. ORMD effect sizes were robust across pre-post correlation coefficients (*r* = 0.3, 0.5, 0.7). Conversely, among RCTs, TTM-based interventions did not produce statistically significant effects on BMI [*k* = 9, g = −0.070, 95% CI (−0.198, 0.058), *p* = 0.285, *I^2^* = 0%], body weight [*k* = 6, g = −0.030, 95% CI (−0.207, 0.147), *p* = 0.742, *I^2^* = 0%], waist circumference [*k* = 4, g = −0.090, 95% CI (−0.281, 0.101), *p* = 0.354, *I^2^* = 31.6%], or self-efficacy [*k* = 3, g = 0.778, 95% CI (−0.077, 1.633), *p* = 0.074, *I^2^* = 92.3%]. For ORMD BMI, subgroup analyses identified intervention format (education plus exercise vs. education only, *p* = 0.0002) and age group (<18 vs. ≥18 years, *p* = 0.035) as significant moderators. Meta-regression showed that baseline BMI, age, number of sessions, and total intervention hours did not significantly moderate the effects of BMI (all *p* > 0.05). Publication bias was not detected for most outcomes, except for RCT weight (Egger’s *p* = 0.029). Leave-one-out sensitivity analyses confirmed the robustness of all pooled estimates.

**Conclusion:**

TTM-based interventions showed significant improvements in BMI, body weight, waist circumference, and self-efficacy when evaluated using ORMDs, with effects robust to assumptions about pre-post correlation. However, these improvements were not statistically significant under the more conservative between-group comparisons of RCTs. The discrepancy highlights the importance of study design in evaluating behavioral interventions. ORMDs may capture within-person change that RCT between-group contrasts fail to detect, but are also susceptible to expectancy and maturation effects. Future research should prioritize well-controlled RCTs with adequate blinding and intention-to-treat analyses, and interventions combining nutritional education with exercise are recommended for optimal BMI reduction.

**Systematic review registration:**

PROSPERO, CRD42024620040.

## 1 Introduction

Overweight and obesity have become major global public health challenges (1). Studies indicate that 2.2 billion adults worldwide are overweight (BMI ≥ 25 kg/m^2^) or obese (BMI ≥ 30 kg/m^2^), accounting for 42.3% of the global adult population; among 430 million children and adolescents (aged 5–19 years), 22.4% are overweight or obese. It is projected that by 2035, the number of overweight or obese adults will rise to 3.3 billion, and the number of overweight or obese children and adolescents will increase to 770 million (2). The countries with the highest prevalence of overweight and obesity are the United States and China. In 2023, 41.9% of American adults were classified as obese (1), while in China, the corresponding figure reached approximately 50% (3). Overweight and obesity not only impose serious health burdens on individuals—including elevated risks of diabetes, stroke, coronary heart disease, and cancer—but also place substantial economic burdens on society. Global obesity treatment costs are projected to reach $4.3 trillion by 2035 (2). The pathogenesis of obesity and overweight is complex. Setting aside genetic factors, the primary cause lies in an imbalance between energy intake and expenditure: excessive caloric consumption coupled with insufficient physical activity leads to weight gain (4). Common measures of overweight and obesity include BMI [calculated as weight (kg)/height (2) (m^2^)] and waist circumference, which reflects abdominal adiposity (central obesity) and is closely associated with visceral fat accumulation, serving as an important predictor of cardiovascular disease (5). Therefore, effective interventions are needed to reduce BMI, weight, and waist circumference in overweight and obese populations.

Among theory-based interventions for modifying unhealthy behaviors, those grounded in the transtheoretical model (TTM) are effective (6, 7). The TTM was developed by American psychologists Prochaska and DiClemente (8). Its core constructs include stages of change, processes of change, decisional balance, self-efficacy, and temptation. Evidence indicates that TTM-based interventions can significantly promote health behavior change by enhancing individuals’ self-efficacy, which in turn positively affects weight, BMI, and waist circumference (9, 10). Within the TTM framework, both online and offline interventions have proven effective in reducing BMI and improving other health indicators among overweight or obese individuals. Online interventions primarily involve email (11) and telephone-based approaches (12, 13), whereas offline modalities include seminars (14) and face-to-face education (15, 16); typically, these two formats are used in combination. Baysal’s (17) study demonstrated that, compared to the control group, obese women in the experimental group exhibited significant reductions in BMI (27.92 ± 1.51 vs. 26.00 ± 1.80, *p* = 0.00) and improved self-efficacy scores following a 10-week offline TTM-based educational intervention with 6 months of telephone follow-up. Menezes’s (14) research similarly showed significant improvements in weight, BMI, and waist circumference in the TTM-based intervention group. However, inconsistent findings have been reported: Boff’s study (15) found that although the self-efficacy of obese individuals in the experimental group improved (*p* < 0.05), BMI did not decrease significantly (34.55 ± 4.37 vs. 34.67 ± 5.60, *p* = 0.63).

In 2011, a Cochrane review (18) concluded that TTM-based interventions had limited effects on weight reduction in obese individuals (approximately 2 kg or less). However, combining TTM stages of change with physical activity and dietary interventions yielded more favorable outcomes, particularly in improving waist circumference and self-efficacy. More recent studies further confirm that TTM-based interventions combined with exercise training can significantly reduce BMI while substantially enhancing self-efficacy scores (16, 19). However, given that this systematic review was completed over a decade ago (2011), included a limited number of studies (*k* = 5), and did not conduct a meta-analysis, an updated systematic review with quantitative synthesis is warranted. Another systematic review examined the impact of TTM-based interventions on adolescent nutrient intake and found that these interventions effectively reduced fat intake, increased fruit and vegetable consumption, and significantly promoted progression through the stages of change (20). Regrettably, that review did not explore the effects of the interventions on BMI among overweight or obese adolescents. A further systematic review examined the effects of TTM-based interventions on dietary habits and physical activity, with health-related outcomes, such as BMI, as secondary endpoints (21). The results indicated that such interventions effectively reduced weight, BMI, waist circumference, and body fat percentage. Although this systematic review included both randomized controlled trials (RCTs) and one-group repeated measures designs (ORMDs), no meta-analysis was conducted. Moreover, the review did not restrict the study population (including both overweight or obese individuals and those with comorbidities such as cardiovascular disease and diabetes) and searched only the PubMed and SciELO databases, which may have led to incomplete literature coverage and reduced the credibility of the conclusions.

Intervention studies based on TTM employ diverse research designs. RCTs (11, 12, 22) are considered the gold standard for causal inference, as they minimize confounding through randomization and enable unbiased estimation of between-group treatment effects. ORMDs (23–25), while lacking the rigorous control of RCTs, offer complementary strengths: they capture within-person change trajectories over time, better approximate real-world conditions where randomization may be infeasible, and can detect preliminary signals of effectiveness that inform subsequent trial design. Recognizing that RCTs and ORMDs estimate fundamentally different effect parameters—between-group differences versus within-group changes—and following established methodological guidance, the present review analyzes these two study designs separately to avoid conflating distinct estimands and to enable direct comparison of design-specific effect patterns. This approach aligns with recommendations from recent meta-epidemiological research, which emphasize that combining RCTs and non-randomized studies in the same meta-analytic model can yield misleading summary estimates when design effects are heterogeneous.

Notably, while TTM provides a unifying theoretical framework, its operationalization in intervention research is highly heterogeneous. Across published studies, TTM-based interventions have been delivered through diverse modalities—face-to-face counseling, telephone coaching, SMS and email messaging, printed materials, group seminars, and web-based platforms—and have varied substantially in which TTM constructs (stages of change, processes of change, decisional balance, self-efficacy) are emphasized, whether interventions are stage-matched or uniformly delivered, and whether they target single or multiple behaviors (20, 26). However, the extent to which this heterogeneity moderates intervention effectiveness remains insufficiently examined. Clarifying the role of these moderating factors is essential for identifying the most effective TTM intervention strategies for overweight or obese populations.

The inclusion of both youth and adult populations in this review reflects the trans-developmental applicability of the TTM. The model’s stage-based architecture is theoretically age-invariant: the processes of change (cognitive and behavioral strategies) and the construct of self-efficacy are developmentally adaptable, and TTM-based interventions have been successfully implemented across the full age spectrum—from children and adolescents (13, 20, 27–30) to middle-aged and older adults (8, 17, 23, 24) Restricting the review to a single age stratum would have precluded examination of age as a potential moderator of intervention effectiveness, which is a key aim of the present study. Previous systematic reviews of TTM-based interventions have similarly included both youth and adult samples (18, 21).

Therefore, this study employed a systematic review and meta-analysis methodology, analyzing RCTs and ORMDs separately. The aims were twofold: (1) to quantify the effectiveness of TTM-based interventions on anthropometric outcomes (BMI, weight, and waist circumference) and a psychosocial outcome (self-efficacy) in overweight or obese populations, separately by study design; and (2) to explore potential moderators—including intervention format, age group, intervention duration, intensity, setting, and publication period—that may explain variability in intervention effects across studies. We hope that these findings will provide evidence-based recommendations for clinical practice and future research.

## 2 Methods

This study was conducted in accordance with the Preferred Reporting Items for Systematic Reviews and Meta-Analyses (PRISMA) guidelines (31) and was prospectively registered on PROSPERO (CRD42024620040; https://www.crd.york.ac.uk/PROSPERO/recorddashboard).

### 2.1 Inclusion and exclusion criteria

Inclusion and exclusion criteria were based on the PICOS framework. (1) Participants: Studies were included if participants met the World Health Organization (WHO) definitions for overweight (adults: BMI 25.0–29.9 kg/m^2^; children and adolescents aged 5–19 years: BMI-for-age ≥ + 1 SD and < +2 SD, corresponding to BMI ≥ 85th and < 95th percentile) or obesity (adults: BMI ≥ 30 kg/m^2^; children and adolescents aged 5–19 years: BMI-for-age ≥ + 2 SD, corresponding to BMI ≥ 95th percentile). Studies involving participants with other medical conditions (e.g., diabetes, polycystic ovary syndrome, or mental disorders) were excluded. (2) Interventions: Studies were eligible if interventions were explicitly grounded in TTM theory, without restrictions on intervention format, duration, or delivery modality; studies using other theoretical frameworks (e.g., Social Cognitive Theory, Theory of Planned Behavior) were excluded. (3) Comparators (for RCTs): Studies in which the control group received usual care were included; studies lacking any comparator were excluded. (4) Outcomes: The primary outcome of this review was BMI, as pre-specified in the PROSPERO protocol (CRD42024620040); secondary outcomes included weight, waist circumference, and self-efficacy. Studies were included if they reported BMI data alongside at least one secondary outcome. Studies that reported weight, waist circumference, or self-efficacy but did not report BMI—for example, because BMI was not calculated from available height and weight measurements—were excluded. (5) Study design: Only RCTs and ORMDs were eligible; conference abstracts and theses were excluded. (6) Language: Only studies published in English or Chinese were included.

### 2.2 Literature search strategy

A comprehensive search was conducted across six databases—CNKI, Cochrane Library (including Embase, ClinicalTrials.gov, and ICTRP), EBSCO (ASP, PsycARTICLES, PsycINFO, SPORTDiscus with Full Text), PubMed, Web of Science, and Scopus—from database inception through March 30, 2025. Search terms included: “transtheoretical,” “stages of change,” “psychological model,” “health education,” “obesity,” “overweight,” “weight loss,” “weight control,” “weight management,” “unhealthy weight,” and “body mass index,” along with their corresponding Chinese terms. Table 1 presents the detailed search strategy using PubMed as an example; strategies for other databases followed the same logic.

**Table 1:** Search strategy (PubMed database example).

Obesity or overweight	
Subject terms (MeSH)	AND
	health education
Free word	“transtheoretical model” OR “stages of change” OR “psychological model” OR “trans-theoretical model”
	AND
	“weight loss” OR “weight control” OR weight management” OR “unhealthy weight” OR “body mass index”

### 2.3 Literature screening

All retrieved records were imported into EndNote (version X9) for duplicate removal. One researcher (DJH) performed the initial deduplication. Subsequently, two researchers (DJH and LWJ) independently screened titles and abstracts against the eligibility criteria. Full texts of studies passing the initial screening were then independently assessed by the same two researchers. Discrepancies at any stage were resolved through discussion with a third researcher (LT) to ensure objectivity of the screening process.

### 2.4 Data extraction and effect size calculation

Two researchers (DJH and LWJ) independently extracted data using a pre-specified, standardized Excel form. Extracted information included: first author (year of publication), country or region, participant characteristics (sample size, age, sex distribution), TTM intervention format (nutritional education alone or nutritional education plus exercise, where nutritional education encompasses seminars, conferences, and personalized counseling), intervention details, total duration, control group procedures (for RCTs), and outcome data. All extracted data were verified by the third researcher (LT). Disagreements among researchers were resolved through consensus discussion. For studies that did not report outcomes as mean ± SD, we attempted to contact the corresponding or first author to obtain the necessary data; if the required data could not be obtained, the study was excluded.

Given the inclusion of both RCTs and ORMDs, we extracted post-test means, standard deviations, and sample sizes for both experimental and control groups from RCTs, as well as pre-test and post-test means, standard deviations, and sample sizes from ORMDs. All data were uniformly converted into Hedges’ g effect sizes and their standard errors. For RCTs, effect sizes were calculated as the standardized mean difference between experimental and control groups at post-test, applying the exact Hedges’ g bias correction (20). For ORMDs, within-group pre-post effect sizes were computed following Borenstein’s method (27, 28), which requires an estimate of the pre-post correlation coefficient (r). Because individual studies did not report this correlation, we conducted sensitivity analyses with *r* = 0.3, 0.5, and 0.7, and used *r* = 0.5 as the primary estimate (27). All effect size calculations were verified against Comprehensive Meta-Analysis (CMA) 3 software to ensure accuracy.

### 2.5 Risk of Bias assessment

For RCTs, two researchers (DJH and LWJ) independently assessed risk of bias using the Cochrane RoB 1.0 tool across seven domains: (1) random sequence generation, (2) allocation concealment, (3) blinding of participants and personnel, (4) blinding of outcome assessors, (5) incomplete outcome data, (6) selective reporting, and (7) other sources of bias. For ORMDs, the ROBINS-I tool was used to evaluate the following seven domains: (1) confounding, (2) selection of participants, (3) classification of interventions, (4) deviations from intended interventions, (5) missing data, (6) measurement of outcomes, and (7) selection of reported results (29). Any disagreements were resolved through consultation with a third researcher (LT).

### 2.6 Statistical analysis

All statistical analyses were conducted using the meta (version 8.0.2) and metafor (version 4.8.0) packages in R (version 4.4.3). Hedges’ g (bias-corrected using an exact formula) served as the effect size measure, with clinical significance thresholds defined as: 0.2 (small), 0.5 (moderate), and 0.8 (large) (32). The inverse-variance weighted method was used to pool effect sizes and calculate 95% confidence intervals. Because RCTs and ORMDs estimate fundamentally different effect parameters (between-group vs. within-group changes), meta-analyses were conducted separately for each study design. A random-effects model with the restricted maximum likelihood (REML) estimator was employed to account for expected heterogeneity across studies. Heterogeneity was assessed using the *I*^2^ statistic (*I*^2^ ≥ 50% indicating substantial heterogeneity) (33) and the *Q*-test. Prediction intervals (PI) were calculated using the t-distribution method to estimate the range within which the true effect of a future study is expected to fall (applicable when k ≥ 3) (34).

The rationale for each subgroup cutoff was as follows. The age-group threshold (<18 vs. ≥18 years) reflects the legal and developmental distinction between pediatric and adult populations, which has implications for BMI assessment (WHO growth standards vs. adult BMI thresholds), intervention delivery (education vs. education + sport), and ethical considerations. The intervention duration cutoff (<6 vs. ≥6 months) was chosen based on the TTM’s theoretical timeline for stage progression: empirical evidence suggests that the minimum time required for meaningful progression through the stages of change from pre contemplation to action is approximately 3–6 months (8, 35), making 6 months a theoretically grounded inflection point at which sustained behavioral enactment becomes more likely. The publication period cutoff (>10 years ago vs. within 10 years) was selected using the landmark Cochrane review by Tuah et al. (2011) (18) as a chronological benchmark. Studies published before 2015 (i.e., more than 10 years before the present review) were conducted before the widespread adoption of digital health technologies and reflect earlier-generation TTM interventions. A 10-year window is commonly used in meta-epidemiological research to distinguish older from contemporary studies, as secular trends in intervention design and reporting standards can introduce temporal confounding.

Publication bias was assessed using contour-enhanced funnel plots (36) and Egger’s regression test (37) (*p* > 0.05, indicating no significant asymmetry). Robustness of the primary findings was evaluated through leave-one-out sensitivity analyses, in which each study was sequentially excluded and pooled effects re-estimated. Additionally, for ORMDs, the sensitivity of pooled estimates to assumptions about the pre-post correlation was examined by repeating all analyses with *r* = 0.3 and *r* = 0.7. All statistical tests were two-tailed with *α* = 0.05.

## 3 Results

### 3.1 Literature screening

**Figure 1:**
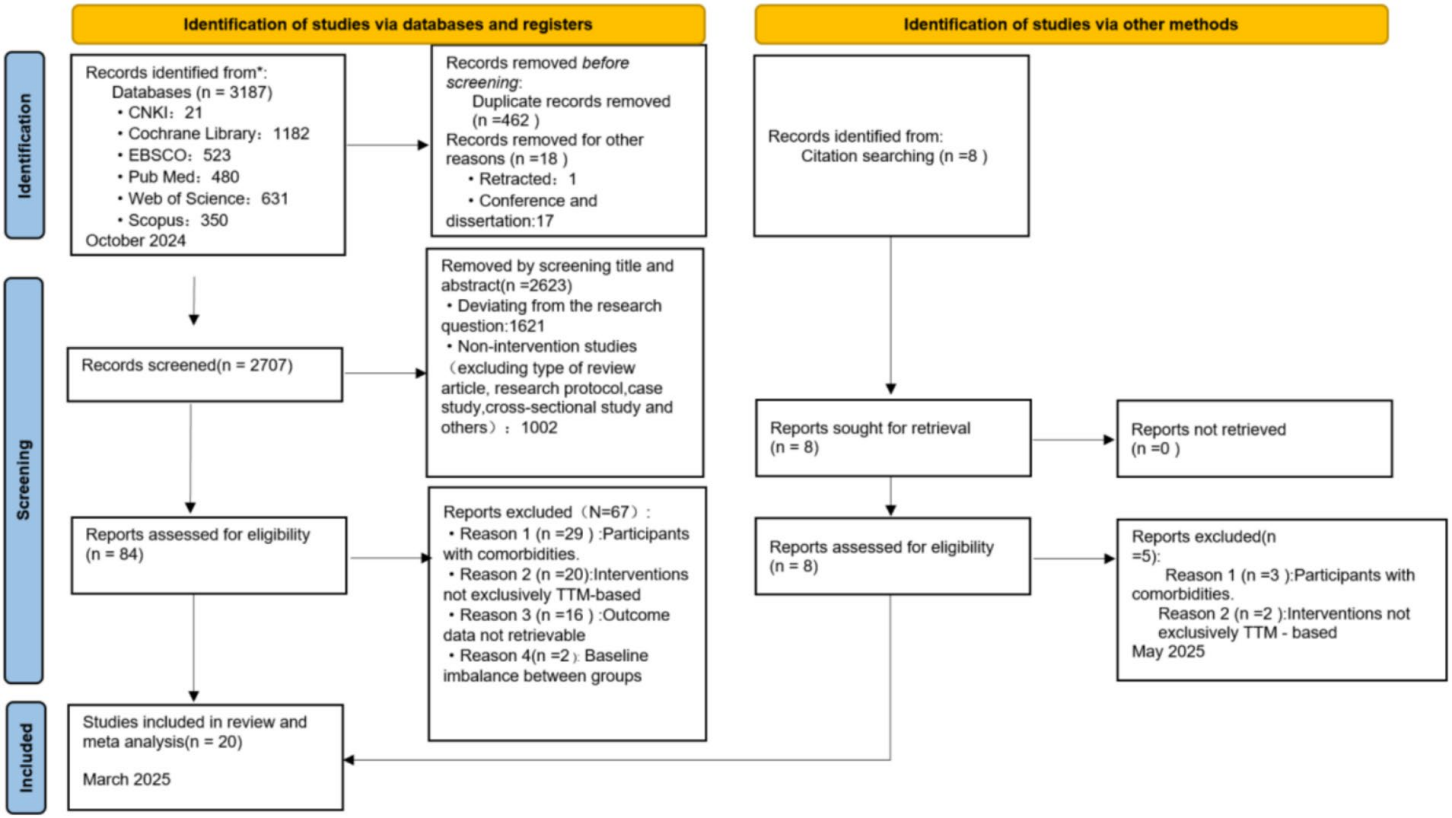
PRISMA flow diagram.

The systematic search across six databases yielded the following results: CNKI (*n* = 21), Cochrane Library (*n* = 1,182), EBSCO (*n* = 523), PubMed (*n* = 480), Web of Science (*n* = 631), and Scopus (*n* = 350). An additional eight articles were identified through reference list screening, bringing the total to 3,195 records. Following the application of inclusion and exclusion criteria, 20 articles were ultimately included in the meta-analysis. The literature screening process is illustrated in Figure 1.

### 3.2 Characteristics of included studies

The 20 included studies were conducted in China (*k* = 4), the United States (*k* = 4), South Korea (*k* = 2), Brazil (*k* = 2), Iran (*k* = 2), Thailand (*k* = 1), Canada (*k* = 1), Australia (*k* = 1), Malaysia (*k* = 1), Italy (*k* = 1), and Turkey (*k* = 1). Publication dates ranged from 2002 to 2024, spanning 22 years. The total sample comprised 1,736 participants, with mean ages ranging from 8 to 60.4 years. TTM was primarily implemented through lectures, case management, personalized counseling, seminars, educational courses, programs, and workshops, collectively referred to as nutritional education. Additionally, several studies used interventions combining nutritional education with exercise. The number of intervention sessions ranged from 4 to 54; individual session durations ranged from 10 to 12 min to 90 min. Total intervention duration ranged from 3 to 10 months. Detailed characteristics are presented in Tables 2, 3.

**Table 2:** Characteristics of included RCTs.

Author (year)	Country	Subjects characteristics						The application form of TTM	Details of interventions	Duration, including follow-up (months)	Outcome measurement
		Baseline BMI ( $\bar{X;\mathrm{kg}/m}$ ^2^)		Age ( $\bar{X;\text{year}}$ )		Included participants (n)					
		CG	IG	CG	IG	CG	IG				
Karintrakul et al. (37)	Thailand	26.98	26.97	32.34	33.32	19^a^	22^a^	nutrition education	Three face-to-face visits, 30–45 min per visit, and 2 weeks per visit by telephone.	6	①②③
Crabtree et al. (22)	America	30.8	31.1	11.2	10.9	10 Men 5	9 Men 3	nutrition education	1 week per session of telephone intervention; 4 weeks per session of face-to-face intervention.	3	①②
Menezes et al. (21)	Brazil	27.7	28.1	60.4	55.9	38^a^	59^a^	nutrition education	A total of 54 times, each 70 min.	6	①②
Ham et al. (27)	Korea	24.22	24.35	10.26	10.77	27 Men 15	48 Men 27	Nutrition education + exercise	Nutrition education: 8 times; Rope skipping: 12 times, each 60 min.	3	①④
Boff et al. (15)	Brazil	35.99	34.74	16.46	16.42	70 Men 15	65 Men 21	nutrition education	A total of 12 times, once a week, each time 1.5 h.	3	①④
Nasser et al. (12)	Canada	30	30	51	51	70 Men 38	71 Men 33	nutrition education	Four face-to-face visits, each 90 min; three outpatient follow-up visits, each 40–45 min; three telephone follow-up visits, each 10–15 min.	10	①②③
Partridge et al. (11)	Australia	27.1	27.3	27.2	28.1	125 Men 46	123 Men 50	nutrition education	8 SMS messages per week; 1 email per week; 5 personalized phone calls.	3	①
Marziyeh et al. (45)	Iran	29.10	30.57	39.18	38.68	78^a^	74^a^	nutrition education	A total of 5 times, each 60 min.	3	①②③
Yusop (16)	Malaysia	3.4	3.2	9.8		20	20	Nutrition education + exercise	Nutritional education: once a week, 30 min each time; Aerobic exercise course: 3 times, each 2 h; Health food preparation activities: 1 time; Parent “Share Care” meeting: 1 time.	6	

CG, control group; IG, intervention group; ①, BMI; ②, weight; ③, waist circumference; ④, self-efficacy; ^a^ The sample represented all women.

**Table 3:** Characteristics of included ORMDs.

Author (year)	Country	Subjects characteristics					The application form of TTM	Details of interventions	Duration, including follow-up (months)	Outcome measurement
		Baseline BMI (Mean±SD)		Age (Mean±SD)		Included participants (n)				
		Mean	SD	Mean	SD					
Zhu Xiaofang et al. (28)	China	23.78	1.52	11.72	1.44	85 (47 Men)	Nutrition education + exercise	Nutrition education: motivational interview for about 20 min; telephone follow-up during the maintenance phase, twice/month; Exercise: Provide exercise supervision and guidance.	6	①
Li Xin et al. (29)	China	26.12	2.82	14.0	0.7	78 (46 Men)	nutrition education	The health brochure takes about 25 min to complete.	6	①④
Zhang Xueyan et al. (13)	China	23.35	1.86	9.6	1.22	81 (54 Men)	nutrition education	Follow-up by telephone 2–3 times/month, each 15–20 min.	6	①
Yang Jian et al. (30)	China	25.75	1.69	11.5	1.75	34 (19 Men)	Nutrition education + exercise	Each session is about 20 min of one-on-one counseling and motivational interview, 2–3 times per month. Exercise: Provide exercise supervision and guidance.	4	①④
Youngho Kim et al. (19)	Korea	29.44	2.38	48.02	5.77	^a^33	Nutrition education + exercise	Nutrition education: 2 lectures per week, 50 min each; Exercise: 3 times/week, 60 min each time.	4	①④
Hormoz Sanaeinasab et al. (54)	Iran	32.5	5.2	About 35 years old	/	48 (48 Men)	nutrition education	A total of 10 times, each 1 h	4	①②③④
Ickes et al. (55)	America	35.7	4.3	25.8	7.5	18 (Men)	nutrition education	260 min group training sessions per week; participate in at least 1 personalized meeting and 2 diet training sessions.	3.5	①④
Buratta Livia et al. (25)	Italy	33.24	4.64	51.49	11.04	100 (51 Men)	Nutrition education + exercise	Nutrition education: 8 times; Structured exercise: 2 times per week, a total of 26 times	3	①②③
Topp et al. (40)	America	21.1	6.2	8	1.82	49 (49% Men)	Nutrition education + exercise	Nutrition education: 1 day per week, including 45 min of nutrition classes (by age group); Exercise: 2 days per week, including flexibility, resistance training, and track and field training.	3.5	①②
Riebe et al. (43)	America	32.4	3.8	50.2	9.2	129 (22% Men)	Nutrition education + exercise	First 3 months: 2 times per week, 2 h each time (1 h of behavior/diet guidance + 1 h of exercise). Last 3 months: 1 time per week in the first month, then once every two weeks, a total of 8 times.	6	①②
Gereklioglu et al. (24)	Turkey	34.2	6.8	47.3	12.8	133 (38.3%) Men	Nutrition education + exercise	5 days per week, including a 45–min walk each day	3	①②

①, BMI; ②, weight; ③, waist circumference; ④, self-efficacy; ^a^ the sample was all women.

The 20 included studies operationalized TTM-based interventions heterogeneously, varying in delivery modality (face-to-face, telephone, SMS/email, group-based, or combined), targeted TTM constructs, and intervention format (nutritional education alone, *n* = 11; nutritional education plus exercise, *n* = 9). This multidimensional diversity warranted the subgroup and meta-regression analyses reported below.

### 3.3 Risk of Bias in included studies

**Figure 2:**
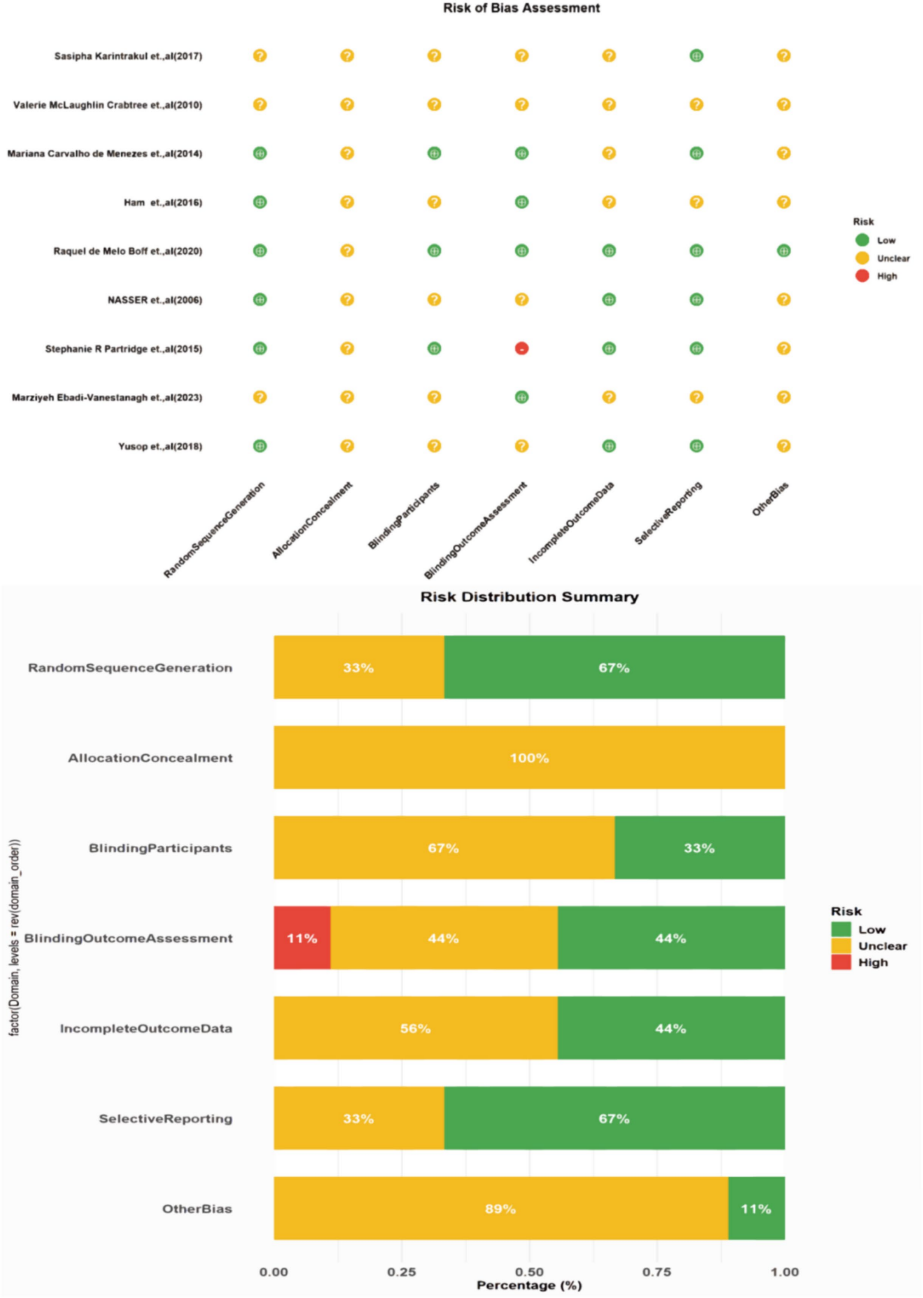
Risk of bias summary: Cochrane RoB tool for RCTs.

**Figure 3:**
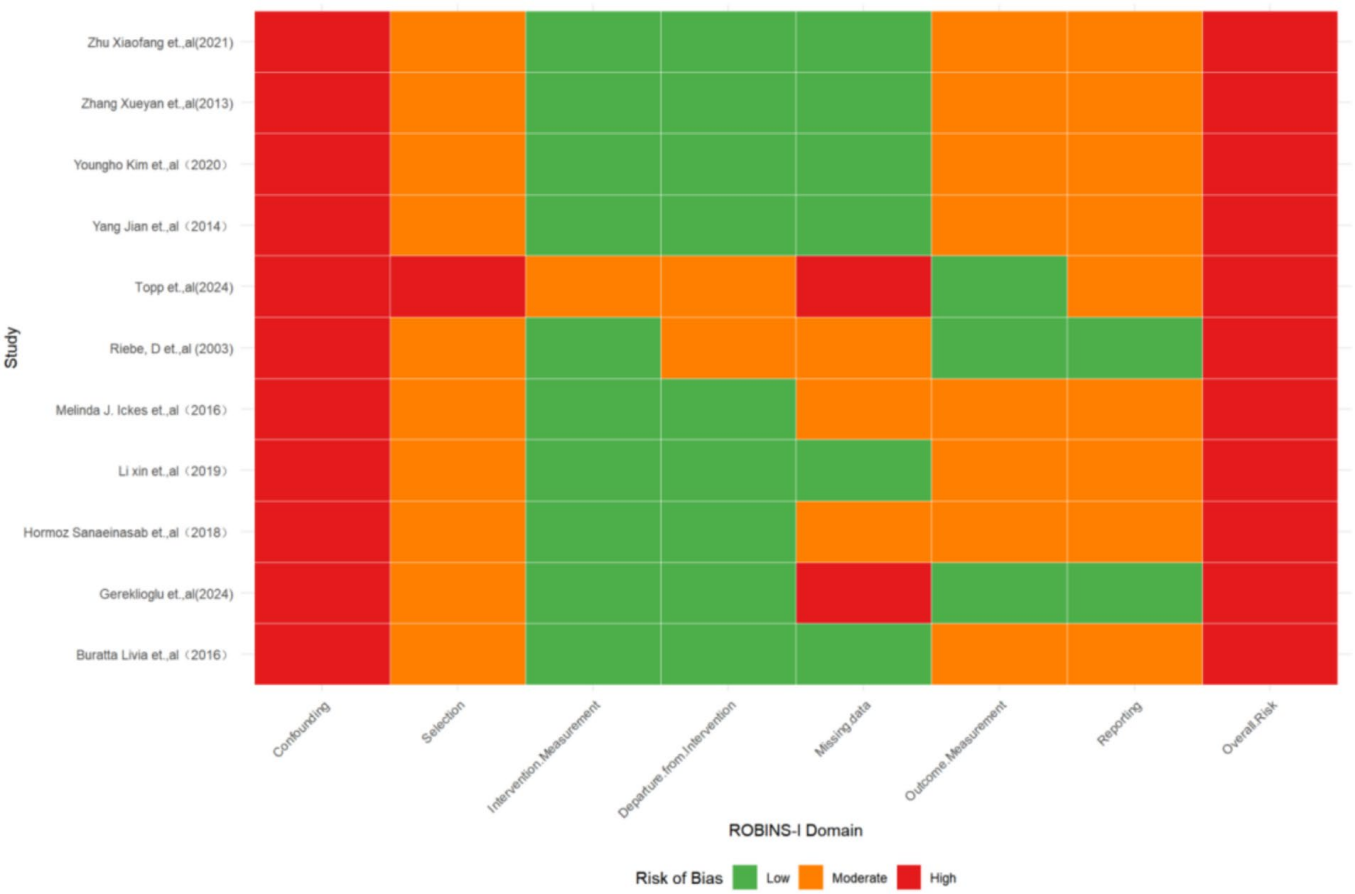
Risk of bias summary: ROBINS-I tool for ORMDs.

Among the 9 included RCTs, one study was rated as low risk of bias (15), one as high risk (11), and the remaining studies were rated as having unclear risk. High and unclear risks were primarily associated with allocation concealment, blinding, and other potential sources of bias, such as low intervention adherence; no study reported conducting an intention-to-treat (ITT) analysis (see Figure 2). All 11 ORMDs were rated as having a high risk of bias due to the absence of control for confounding factors. According to the ROBINS-I guidance in the Cochrane Handbook, if any domain is rated as high risk, the overall study is considered to have a high risk of bias (see Figure 3).

### 3.4 Meta-analysis results

**Figure 4:**
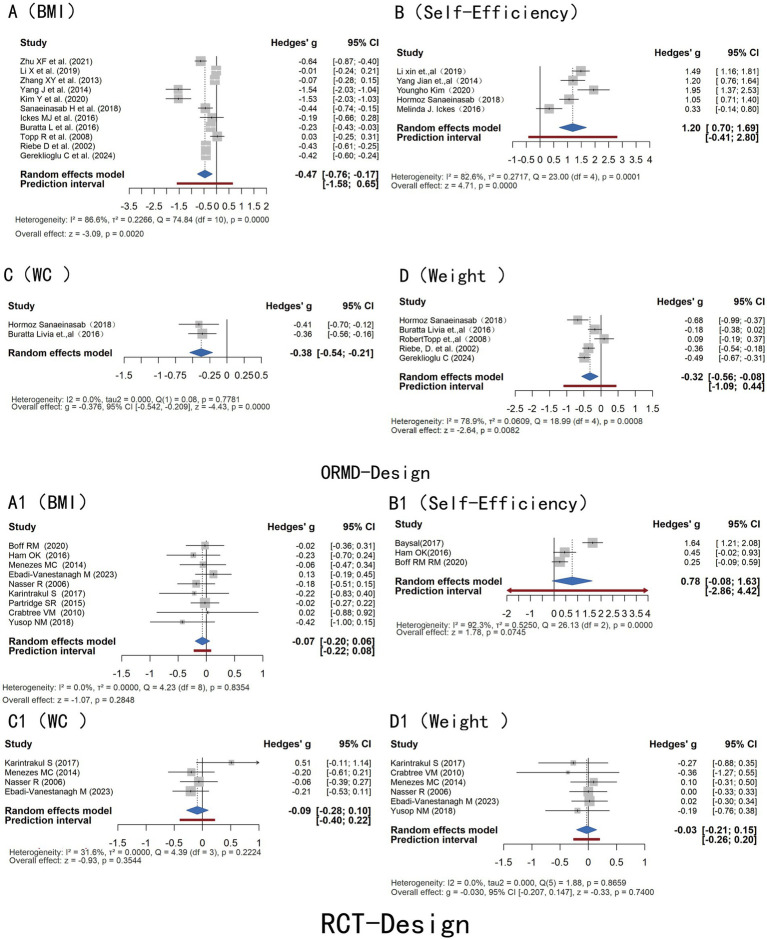
Forest plots for ORMD **(A–D)** and RCT **(A1–D1)** meta-analyses across four outcomes.

Meta-analyses were performed separately for ORMDs and RCTs across four outcomes: BMI, body weight, waist circumference, and self-efficacy. A summary of pooled results is presented in Table 4 (ORMDs) and Table 5 (RCTs). Forest plots displaying individual study effects and pooled estimates are presented in Figure 4.

**Table 4:** ORMD BMI subgroup analysis.

Variable	Subgroup	*k*	Hedge’s g	95% CI	*I*^2^ (%)	*τ* ^2^	Q_b	df	p_b
Age group	<18 years	5	−0.402	[−0.808, 0.005]	91.5	0.192	4.43	1	0.0354*
	≥18 years	6	−0.482	[−0.709, −0.255]	78.4	0.057			
Location	Hospital	5	−0.315	[−0.529, −0.100]	81.9	0.049	2.93	2	0.2306
	Other	2	−0.309	[−0.513, −0.105]	29.8	0.007			
	School	4	−0.792	[−1.641, 0.058]	93.8	0.700			
Intensity	High	5	−0.463	[−0.793, −0.134]	86.4	0.114	4.60	2	0.1001
	Low	2	−0.219	[−0.640, 0.203]	81.0	0.075			
	Medium	4	−0.564	[−1.002, −0.125]	91.6	0.177			
Intervention format	Education + exercise	7	−0.607	[−0.892, −0.323]	89.0	0.124	14.29	1	0.0002***
	Education only	4	−0.157	[−0.351, 0.038]	48.8	0.019			
Publication period	>10 years ago	4	−0.450	[−0.876, −0.025]	91.8	0.166	2.33	1	0.1273
	Within 10 years	7	−0.451	[−0.702, −0.200]	83.4	0.090			
Duration	<6 months	7	−0.411	[−0.637, −0.186]	81.1	0.069	0.53	1	0.4662
	≥6 months	4	−0.518	[−1.012, −0.025]	93.0	0.230			

*p < 0.05, ** *p* < 0.01, *** *p* < 0.001.

**Table 5:** Egger’s regression test for funnel plot asymmetry.

Design	Outcome	k	Intercept	SE	95% CI	*t*	*p*
ORMD	BMI	11	−3.8973	2.4081	[−8.6171, 0.8225]	−1.62	0.1400
ORMD	Self-efficacy	5	−0.3709	6.0335	[−12.1966, 11.4548]	−0.06	0.9548
ORMD	Body weight	5	1.2721	5.1840	[−8.8886, 11.4327]	0.25	0.8220
ORMD	Waist circumference	2	N/A	N/A	N/A	N/A	N/A
RCT	BMI	9	−0.9773	0.6989	[−2.3471, 0.3926]	−1.40	0.2047
RCT	Self-efficacy	3	9.8739	17.5248	[−24.4747, 44.2226]	0.56	0.6734
RCT	Body weight	6	−1.4828	0.4427	[−2.3506, −0.6151]	−3.35	0.0286*
RCT	Waist circumference	4	3.8214	1.7224	[0.4455, 7.1974]	2.22	0.1567

NA indicates that the number of studies was insufficient to perform Egger’s test.

#### 3.4.1 One-group repeated measures designs (ORMDs)

The meta-analysis of 11 ORMDs for BMI showed that TTM-based interventions significantly reduced BMI [*g* = −0.467, 95% CI (−0.763, −0.171), *p* = 0.002, *I^2^* = 86.6%, *τ*^2^ = 0.226], with substantial heterogeneity. The prediction interval was [−1.596, 0.663], indicating that the effect may not generalize to all future settings.

**Figure 5:**
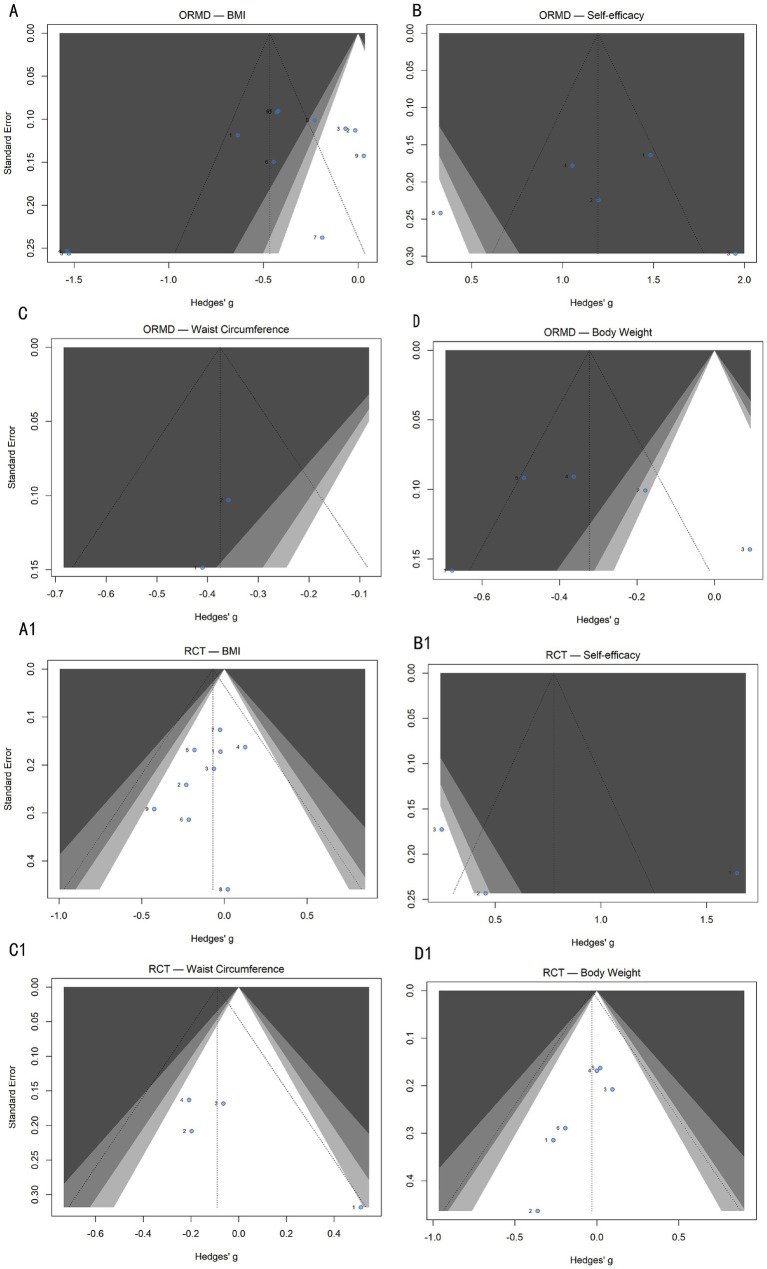
Contour-enhanced funnel plots for ORMD **(A–D)** and RCT **(A1–D1)** meta-analyses across four outcomes.

For body weight, five ORMDs yielded a significant pooled effect [*g* = −0.323, 95% CI (−0.563, −0.084), *p* = 0.008, *I^2^* = 78.9%, *τ*^2^ = 0.061], with prediction interval [−1.198, 0.552]. For waist circumference, two ORMDs produced a significant effect [g = −0.375, 95% CI (−0.542, −0.209), *p* < 0.001, *I^2^* = 0%], though prediction intervals were not computed due to insufficient studies (*k* < 3). For self-efficacy, five ORMDs showed a large and significant improvement [*g* = 1.195, 95% CI (0.699, 1.692), *p* < 0.001, *I^2^* = 82.6%, *τ*^2^ = 0.271], with a prediction interval [−0.648, 3.039] (Figure 5 Upper A–D).

#### 3.4.2 Randomized controlled trials (RCTs)

The meta-analysis of 9 RCTs on BMI did not detect a statistically significant effect [*g* = −0.070, 95% CI (−0.198, 0.058), *p* = 0.285, *I*^2^ = 0%, *τ*^2^ = 0], with prediction interval [−0.224, 0.084]. For body weight, six RCTs similarly showed no significant effect [*g* = −0.030, 95% CI (−0.207, 0.147), *p* = 0.742, *I*^2^ = 0%], with a prediction interval [−0.280, 0.221]. For waist circumference, four RCTs yielded a non-significant pooled effect [*g* = −0.090, 95% CI (−0.281, 0.101), *p* = 0.354, *I*^2^ = 31.6%], with a prediction interval [−0.509, 0.329]. For self-efficacy, three RCTs showed a large but non-significant effect [*g* = 0.778, 95% CI (−0.077, 1.633), *p* = 0.074, *I*^2^ = 92.3%, *τ*^2^ = 0.525], with a prediction interval [−9.966, 11.522]; the extremely wide prediction interval reflects the small number of studies and high heterogeneity (Figure 5 Lower A1–D1).

### 3.5 Subgroup analysis and Meta-regression for ORMD

#### 3.5.1 Subgroup analysis(BMI)

Subgroup analyses for ORMD BMI outcomes identified two significant moderators (Table 6). First, intervention format significantly moderated the effect (Q_b = 14.29, *p* = 0.0002): interventions combining nutritional education with exercise produced a moderate-to-large effect [*k* = 7, g = −0.607, 95% CI (−0.892, −0.323), *I*^2^ = 89.0%], whereas education-only interventions yielded a small, non-significant effect [k = 4, g = −0.157, 95% CI (−0.351, 0.038), *I*^2^ = 48.8%]. Second, age group was a significant moderator (Q_b = 4.43, *p* = 0.035): both age groups showed significant effects, with similar magnitudes [age < 18 years: *k* = 5, *g* = −0.402, 95% CI (−0.808, 0.005), *I*^2^ = 91.5%; age ≥ 18 years: *k* = 6, *g* = −0.482, 95% CI (−0.709, −0.255), *I*^2^ = 78.4%]. No significant subgroup differences were found for intervention setting (*p* = 0.231), intervention intensity (*p* = 0.100), publication period (*p* = 0.127), or intervention duration (*p* = 0.466). The sex subgroup was excluded from the primary analysis because the Women and Men subgroups each contained only one study (*k* = 1), rendering between-group comparisons unreliable.

**Table 6:** ORMD sensitivity analysis: pooled effect sizes under different pre-post correlation coefficients (r).

Outcome	*k*	*r* = 0.3 g	*r* = 0.3 95% CI	*r* = 0.5 g	*r* = 0.5 95% CI	*r* = 0.7 g	*r* = 0.7 95% CI	g range	Robust?
BMI	11	−0.453	[−0.736, −0.171]	−0.467	[−0.763, −0.171]	−0.479	[−0.788, −0.170]	0.026	Yes
Body weight	5	−0.323	[−0.554, −0.093]	−0.323	[−0.563, −0.084]	−0.323	[−0.571, −0.076]	0.0002	Yes
Waist circumference	2	−0.375	[−0.572, −0.179]	−0.375	[−0.542, −0.209]	−0.375	[−0.504, −0.247]	0.000	Yes
Self-efficacy	5	1.192	[0.707, 1.678]	1.195	[0.699, 1.692]	1.198	[0.691, 1.706]	0.006	Yes

#### 3.5.2 Meta-regression (BMI)

**Figure 6:**
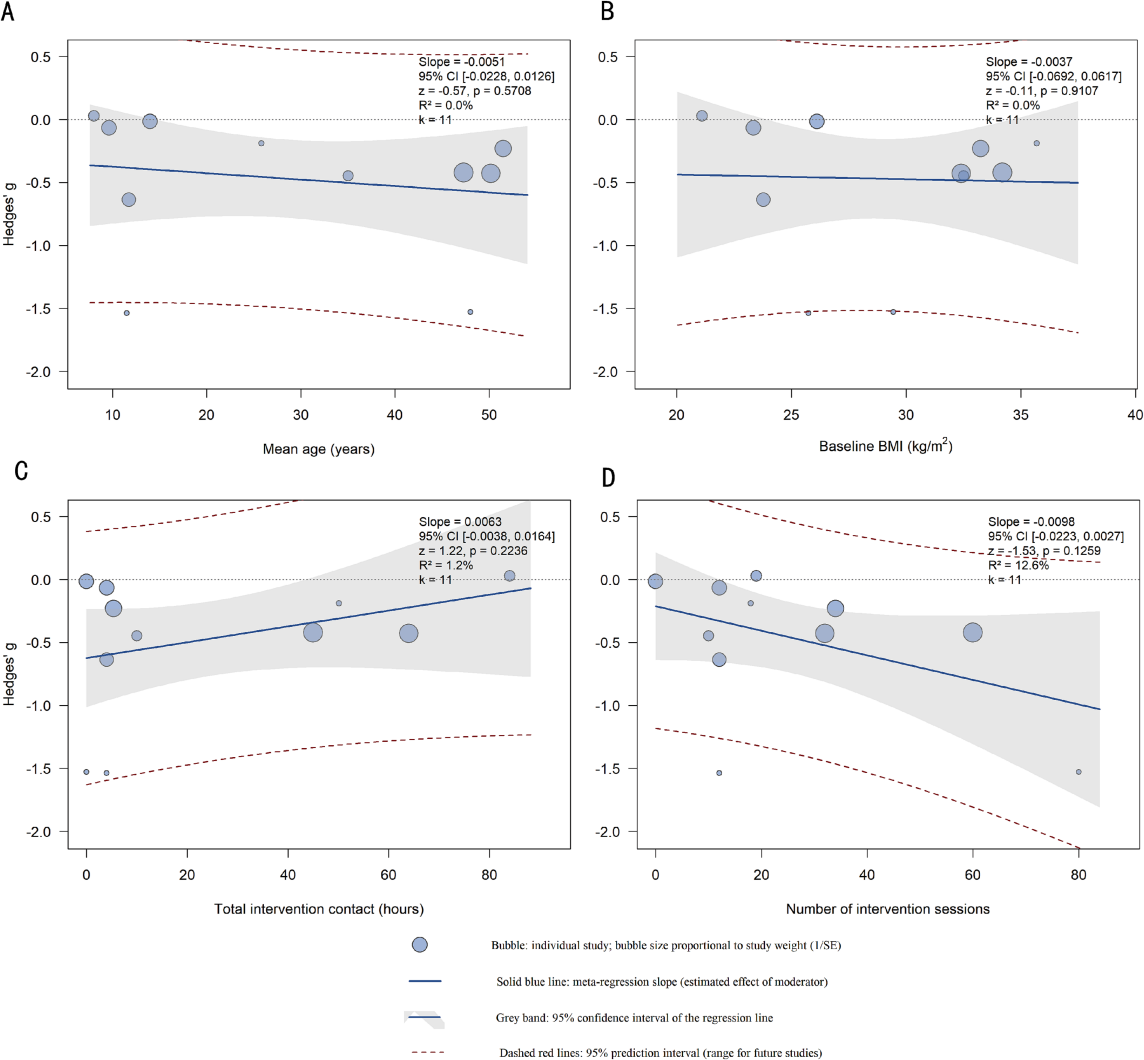
Meta-regression plots for ORMD BMI: **(A)** Mean age, **(B)** Baseline BMI, **(C)** Total intervention hours, **(D)** Number of sessions.

Meta-regression analyses of ORMD BMI outcomes examined four continuous moderators: baseline BMI, mean age, number of intervention sessions, and total intervention hours. None of the continuous moderators significantly predicted BMI effect sizes. Baseline BMI (*β* = −0.004, SE = 0.033, *p* = 0.911, *R*^2^ = 0.0%), mean age (*β* = −0.005, SE = 0.009, *p* = 0.571, *R*^2^ = 0.0%), and total intervention hours (*β* = 0.006, SE = 0.005, *p* = 0.224, *R*^2^ = 1.4%) explained negligible proportions of between-study variance. The number of intervention sessions showed a trend toward greater BMI reduction with more sessions, but this did not reach statistical significance (*β* = −0.010, SE = 0.006, *p* = 0.126, *R*^2^ = 12.6%). Meta-regression bubble plots are presented in Figure 6.

### 3.6 Publication Bias

Funnel plots for all four outcomes under both study designs are presented in Figure 5. Visual inspection suggested approximate symmetry for most outcomes. Egger’s regression test results are summarized in Table 5. For ORMDs, Egger’s test was non-significant for BMI (intercept = −3.90, *p* = 0.140), body weight (intercept = 1.27, *p* = 0.822), and self-efficacy (intercept = −0.37, *p* = 0.955); the test was not applicable for waist circumference (*k* = 2). For RCTs, Egger’s test was non-significant for BMI (intercept = −0.98, *p* = 0.205), waist circumference (intercept = 3.82, *p* = 0.157), and self-efficacy (intercept = 9.87, *p* = 0.673) but reached significance for body weight (intercept = −1.48, *p* = 0.029), suggesting possible small-study effects for this outcome. However, given the small number of studies (*k* = 6 for RCT weight), this result should be interpreted with caution.

### 3.7 Sensitivity analysis

Leave-one-out sensitivity analyses confirmed the robustness of the pooled effect sizes for all outcomes under both study designs. For ORMDs, sequential exclusion of individual studies did not substantively alter the pooled estimates: the recalculated g values for BMI ranged from −0.370 to −0.545, and all remained statistically significant (*p* < 0.05). Similarly, pooled estimates for BMI from RCT remained within the range of −0.115 to −0.022 across all leave-one-out iterations, and none reached statistical significance. Sensitivity analyses for weight, waist circumference, and self-efficacy under both designs likewise demonstrated stability (see Supplementary Material S1).

Sensitivity analyses for the pre-post correlation coefficient (r) in ORMDs confirmed that the pooled effect estimates were highly robust to assumptions about r. For BMI, g ranged from −0.453 (*r* = 0.3) to −0.479 (*r* = 0.7), a difference of only 0.026. For weight, the g range was 0.0002; for waist circumference, 0.000; and for self-efficacy, 0.006. These negligible variations indicate that the choice of r within the conventional range of 0.3–0.7 has minimal impact on the conclusions (Table 6).

## 4 Discussion

This meta-analysis synthesized data from 20 independent studies to evaluate the effects of TTM-based interventions on BMI and other health outcomes in overweight or obese populations, with separate analyses for ORMDs and RCTs. The results reveal a marked discrepancy between the two study designs: ORMDs showed significant improvements across all four outcomes, whereas RCTs showed no statistically significant effects on any outcome. This design-dependent pattern has important implications for interpreting the evidence base and guiding future research.

The present meta-analysis also revealed substantial heterogeneity in ORMD BMI outcomes (*I*^2^ = 86.6%), which may be partly attributable to the inherent diversity in how TTM was operationalized across studies. Although all interventions shared a common TTM foundation, they differed markedly in delivery modality, emphasis on specific TTM constructs (stages of change, processes of change, decisional balance, and self-efficacy), fidelity to stage-matched delivery, and whether exercise was included as a co-intervention (20, 38). The subgroup finding that combined nutrition-plus-exercise interventions outperformed education-only interventions (g: −0.607 vs. −0.157) supports the hypothesis that interventions that engage a broader range of TTM constructs—particularly those reinforcing self-efficacy through experiential mastery—yield greater BMI reductions. This is consistent with the TTM’s theoretical premise that behavioral processes (e.g., counter-conditioning, reinforcement management) become increasingly critical as individuals progress from contemplation to action and maintenance (38). Conversely, interventions relying solely on nutritional education may remain predominantly in the cognitive domain, inadequately supporting the transition from intention to sustained behavioral enactment. This interpretation aligns with Johnson et al. (35), who demonstrated that TTM-based interventions simultaneously targeting multiple behaviors (diet, physical activity, and stress management) achieved greater weight reductions than single-behavior approaches. Future studies should explicitly report which TTM constructs and processes of change are targeted, to enable more precise identification of the active ingredients driving intervention effects (39).

### 4.1 Effects of TTM-based interventions on BMI

The ORMD meta-analysis confirmed that TTM-based interventions significantly reduced BMI (*g* = −0.467, *p* = 0.002, *k* = 11), with a moderate effect size consistent with prior systematic reviews (18, 20). The underlying mechanism involves three processes through which TTM promotes the full trajectory from cognitive awakening to behavioral maintenance: stage-adaptive matching, cognitive-behavioral linkage, and social-environmental coordination (38). Specifically, TTM-based interventions enable overweight or obese individuals to recognize the importance of health and the risks of unhealthy behaviors, motivating them to reduce fat intake, increase fruit and vegetable consumption, engage in physical activity, and ultimately reduce BMI.

However, the RCT meta-analysis did not detect a significant BMI reduction (*g* = −0.070, *p* = 0.285, *k* = 9), with the confidence interval narrowly spanning zero and heterogeneity near zero (*I^2^* = 0%). This null finding deserves careful interpretation. The near-zero *I*^2^ indicates that RCTs produced consistently small effects across diverse populations and settings, suggesting that the lack of significance is not due to heterogeneous effects canceling out but rather reflects a systematically small between-group difference. Several factors may account for this discrepancy between ORMD and RCT findings. First, from a statistical perspective, ORMDs capture within-person change by controlling for individual baseline differences, yielding greater statistical sensitivity but may overestimate true intervention effects due to expectancy effects [e.g., the Hawthorne effect (40)] and maturation. RCTs, through between-group comparisons, control for these threats to internal validity but require larger sample sizes to achieve comparable power. Second, TTM-based interventions are predominantly educational in nature, and the control groups in most included RCTs received some form of usual care or minimal intervention, which may itself produce small benefits, attenuating the between-group contrast. Third, several RCTs reported low intervention adherence, and none conducted ITT analyses, which may further dilute treatment effects in between-group comparisons. These findings underscore the importance of distinguishing between within-group change and between-group difference when evaluating behavioral interventions: the former may overestimate clinical effectiveness, while the latter may underestimate it when interventions are compared against active, albeit minimal, control conditions.

Subgroup analyses of ORMDs identified intervention format and age group as significant moderators. The superiority of combined nutrition-plus-exercise interventions (*g* = −0.607) over education-only interventions (*g* = −0.157, p_b = 0.0002) can be interpreted through multiple mechanisms. From a physiological perspective, the combination generates a metabolic synergistic effect: exercise increases energy expenditure and counteracts the metabolic adaptation induced by caloric restriction alone (e.g., the decline in basal metabolic rate), thus enhancing the efficiency of BMI improvement (41). Exercise may also improve dietary adherence through modulation of appetite-regulating hormones (e.g., ghrelin, GLP-1) (42). From a psychological perspective, TTM emphasizes the staged nature of behavior change and the interplay of core constructs; comprehensive interventions may more fully engage these TTM elements. The immediate sense of accomplishment derived from exercise can bolster self-efficacy, which in turn promotes sustained dietary control; by contrast, the benefits of nutritional education alone often require delayed gratification, leaving adherence vulnerable to frustration and attrition (43). The significant moderating effect of age group (p_b = 0.035) suggests that TTM-based interventions are comparably effective for both youth and adults, though the slightly larger effect in adults (*g* = −0.482) compared to youth (*g* = −0.402) may reflect greater baseline BMI and potentially greater room for improvement in adult populations.

Meta-regression analyses did not identify significant linear associations between BMI effect sizes and continuous moderators (baseline BMI, age, sessions, hours). The number of intervention sessions showed a trend-level association (*β* = −0.010, *p* = 0.126, *R*^2^ = 12.6%), consistent with a dose–response pattern; however, the small number of studies limits statistical power to detect such relationships. The negligible *R*^2^ for baseline BMI (0.0%) and mean age (0.0%) suggests that these factors do not linearly moderate treatment response, though non-linear or threshold effects cannot be ruled out.

### 4.2 Effects on weight and waist circumference

For body weight and waist circumference, the pattern mirrored that of BMI: ORMDs detected significant reductions (weight: *g* = −0.323; WC: *g* = −0.375), whereas RCTs did not (weight: *g* = −0.030; WC: *g* = −0.090). The ORMD findings align with behavior change theory predictions: TTM’s core strength lies in its stage-tailored intervention strategy, whereby designing personalized programs matched to an individual’s current stage of change can significantly improve adherence (44). For individuals in the precontemplation stage, the focus is on cognitive restructuring, whereas for those in the action stage, environmental support is intensified. This differentiated strategy is more effective than traditional one-size-fits-all approaches. Moreover, the self-efficacy and decisional balance constructs emphasized by the TTM may serve as key mediators. In the ORMD analysis, participants’ self-efficacy improved substantially (*g* = 1.195), and other studies have similarly reported that participants’ perceived benefits of healthy eating increased while perceived barriers decreased post-intervention (26, 45)These findings are consistent with Bandura’s social cognitive theory: enhanced self-efficacy strengthens an individual’s confidence in overcoming challenges, while shifts in decisional balance facilitate long-term behavior maintenance through cost–benefit appraisal (46).

The null RCT findings for weight and waist circumference are noteworthy. In several RCTs (21, 22, 37), although some weight-related outcomes decreased in the intervention group, no statistically significant differences were observed between groups. Possible reasons include: (1) control groups receiving usual care may have experienced modest improvements, reducing between-group differences; (2) reductions in body weight and waist circumference may be more directly linked to physical exercise than to nutritional education alone (47); and (3) at present, TTM-based interventions focus predominantly on modifying behavioral stages and enhancing motivation for change, while relatively little attention is paid to effectively guiding individuals to engage in physical exercise once they have transitioned across stages. Few studies explicitly describe how the intervention operationalized physical exercise in terms of type, frequency, and intensity. The significant Egger’s test for RCT weight outcomes (*p* = 0.029) further warrants caution, as it raises the possibility of small-study effects.

### 4.3 Effects on self-efficacy

This meta-analysis integrated self-efficacy data from eight studies (ORMD: *k* = 5; RCT: *k* = 3), confirming that TTM-based interventions can substantially enhance self-efficacy, though the magnitude and precision of the estimates varied markedly by study design. ORMDs showed a large, significant improvement (*g* = 1.195, *p* < 0.001), while RCTs showed a similarly large but non-significant effect with a wide confidence interval [*g* = 0.778, *p* = 0.074, 95% CI (−0.077, 1.633)]. The RCT result is heavily influenced by Baysal (17), which reported an exceptionally large effect (*g* = 1.645) and accounted for 33.2% of the weight. The high heterogeneity (*I*^2^ = 92.3%) and extremely wide prediction interval [−9.966, 11.522] indicate that the self-efficacy effect under RCT conditions is highly variable and imprecisely estimated with only three studies.

Self-efficacy is a central construct in social cognitive theory (48) and is positioned as a key predictor of behavior change within the TTM framework. The present ORMD findings suggest that TTM enhances self-efficacy through a two-stage mechanism: (1) the cognitive restructuring stage (precontemplation → contemplation), in which decisional balance and scenario-based training reduce fear of anticipated failure; and (2) the behavioral reinforcement stage (action → maintenance), in which progressive goal-setting and positive feedback accumulate mastery experiences. This phased and incremental strategy is highly consistent with Bandura’s (46) proposed mechanism of mastery experience as the primary source of self-efficacy. Notably, the improvement in self-efficacy was substantially larger than the changes in anthropometric outcomes, reinforcing the conclusion that TTM-based interventions have particular strength in modifying behavioral intentions. However, translating these enhanced intentions into measurable reductions in BMI, weight, and waist circumference requires additional effective strategies to bridge the intention-behavior gap (49).

The centrality of self-efficacy to TTM-based interventions is further supported by the model’s stage-dependent architecture. Within the TTM, self-efficacy is not a static trait but a dynamic construct that increases progressively as individuals advance from precontemplation through to maintenance (38). This theoretical proposition has received robust empirical support. Andrés et al. (50, 51) used the S-Weight and P-Weight instruments, specifically developed and validated for weight-management populations, demonstrated that self-efficacy scores increase monotonically across the five stages of change, with the largest gains occurring between the contemplation and action stages. Karintrakul and Angkatavanich (37) provided experimental evidence for this mechanism in a clinical context: in their 12-week RCT, TTM-matched individualized nutrition counseling significantly enhanced the Weight Management Action process relative to a non-stage-matched educational control, and this enhancement was accompanied by clinically meaningful reductions in body weight (−1.98 kg) and waist circumference (−5.35 cm). These findings suggest that the large pooled effect on self-efficacy observed in ORMDs (*g* = 1.195) may reflect a genuine mechanism of action: TTM-based interventions are designed to systematically bolster self-efficacy as the primary driver of stage progression, and this mechanism operates across diverse delivery formats and populations. Future studies would benefit from routinely reporting stage-specific self-efficacy scores to enable more direct examination of this theoretical pathway.

### 4.4 Limitations

Despite yielding several relatively robust conclusions, this meta-analysis has important limitations that should be considered when interpreting the findings. (1) Methodological quality: Only one RCT was rated as having a low risk of bias; the majority had unclear allocation concealment and lack of blinding, which may lead to overestimation of intervention effects. However, achieving adequate blinding is inherently challenging in educational program interventions, particularly with respect to participant blinding (52). All included ORMDs lacked adjustment for confounding and were rated as high risk of bias, which limits causal attribution of within-group changes. (2) Completeness of the evidence base: During full-text screening, several studies met the inclusion criteria but did not report BMI and other outcomes as mean ± SD; attempts to contact the authors for these data were unsuccessful. Although these studies reported no significant differences in BMI between the experimental and control groups (26, 53), their exclusion introduces potential reporting bias. Furthermore, our inclusion criterion requiring studies to report BMI may have excluded some TTM-based intervention studies that collected anthropometric data but did not report BMI as an outcome. These studies could have provided supplementary information on related outcomes; their exclusion may contribute to a less complete picture of the broader effects of TTM-based interventions. (3) Limited evidence on long-term effects: Only one study had a follow-up period of 24 months; all others were shorter than one year, precluding assessment of the durability of TTM-based intervention effects. (4) Uncertainty in ORMD effect size estimation: Due to the unavailability of raw data, the pre-post correlation coefficient was set at *r* = 0.5 for all ORMD effect size calculations. However, sensitivity analyses at *r* = 0.3, 0.5, and 0.7 demonstrated that the pooled estimates are robust to this assumption (g range ≤ 0.026 across all outcomes), substantially mitigating this concern. (5) Small number of studies in certain subgroups and outcomes: The waist circumference analysis for ORMDs included only two studies, and the RCT self-efficacy analysis included only three studies with extremely wide prediction intervals. Subgroup and meta-regression findings should be interpreted with caution, given the limited degrees of freedom. (6) Inability to isolate active ingredients: Although the subgroup analysis identified intervention format as a significant moderator, the included studies did not consistently report which specific TTM constructs were targeted, precluding more granular analysis of the mechanisms through which TTM-based interventions exert their effects.

## 5 Conclusion

TTM-based interventions produce significant improvements in BMI, body weight, waist circumference, and self-efficacy among overweight or obese populations when evaluated using ORMDs, with effects that are robust to assumptions about the pre-post correlation. However, these improvements were not statistically significant in between-group comparisons from RCTs. Several implications follow from these findings. First, the discrepancy between ORMD and RCT estimates underscores the critical importance of study design in evaluating behavioral interventions: within-group changes may overestimate clinical effectiveness due to uncontrolled confounds, whereas between-group differences may underestimate benefits when control conditions receive active albeit minimal interventions. Second, when the goal is BMI reduction, interventions combining nutritional education with exercise are recommended, as they produced substantially larger effects than education-only interventions (*g*: −0.607 vs. −0.157). Third, future RCTs should prioritize: (a) adequate sample sizes to detect the small-to-moderate between-group effects suggested by the present evidence; (b) standardized reporting of intervention components, including explicit description of which TTM constructs are targeted; (c) intention-to-treat analyses to account for intervention non-adherence and attrition; (d) longer follow-up periods to assess the durability of TTM-based intervention effects; and (e) inclusion of specific physical exercise protocols, particularly as participants transition into the action and maintenance stages of change. Finally, when ORMDs are employed, researchers should strengthen the control of confounding factors and report the pre-post correlation coefficient to enable more rigorous evaluation by other investigators.

## Data Availability

The original contributions presented in the study are included in the article/Supplementary material, further inquiries can be directed to the corresponding author.
