# Supplementary material for: Effects of transtheoretical model-based interventions on body mass index and other health outcomes in overweight or obese populations: a systematic review and meta-analysis
Source: Front Public Health. 2026 Jun 22;14:1832812. doi: 10.3389/fpubh.2026.1832812 (PMC13335073; doi:10.3389/fpubh.2026.1832812)
Supplement: Supplementary file 1 [file Data_Sheet_1.pdf]

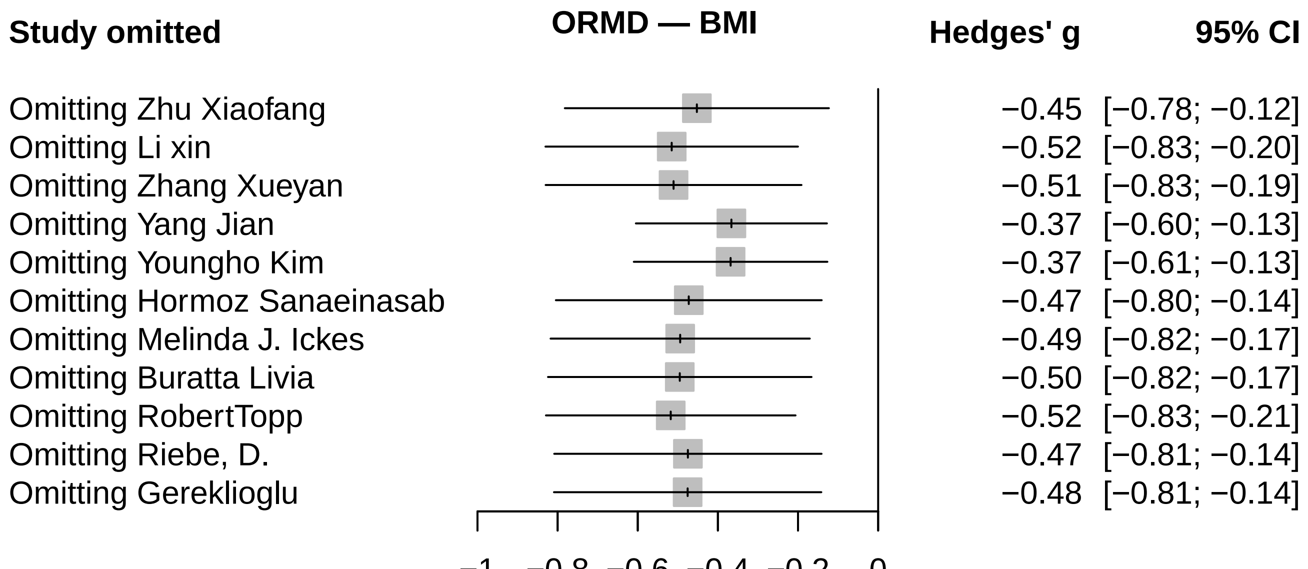
Study omitted
ORMD — BMI
Hedges' g
95% CI
Omitting Zhu Xiaofang −0.45 [−0.78; −0.12]
Omitting Li xin −0.52 [−0.83; −0.20]
Omitting Zhang Xueyan −0.51 [−0.83; −0.19]
Omitting Yang Jian −0.37 [−0.60; −0.13]
Omitting Youngho Kim −0.37 [−0.61; −0.13]
Omitting Hormoz Sanaeinasab −0.47 [−0.80; −0.14]
Omitting Melinda J. Ickes −0.49 [−0.82; −0.17]
Omitting Buratta Livia −0.50 [−0.82; −0.17]
Omitting RobertTopp −0.52 [−0.83; −0.21]
Omitting Riebe, D. −0.47 [−0.81; −0.14]
Omitting Gereklioglu −0.48 [−0.81; −0.14]
−1 −0.8 −0.6 −0.4 −0.2 0

ORMD — Self−efficacy

Study omitted	Hedges' g	95% CI
Omitting Li xin et.,al	1.12	[0.49; 1.74]
Omitting Yang Jian et.,al	1.20	[0.55; 1.85]
Omitting Youngho Kim	1.04	[0.57; 1.50]
Omitting Hormoz Sanaeinasab	1.23	[0.58; 1.89]
Omitting Melinda J. Ickes	1.38	[1.04; 1.72]

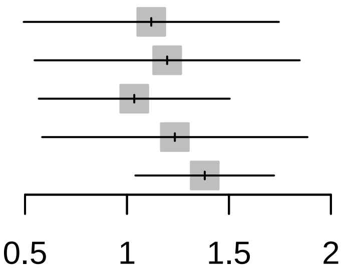

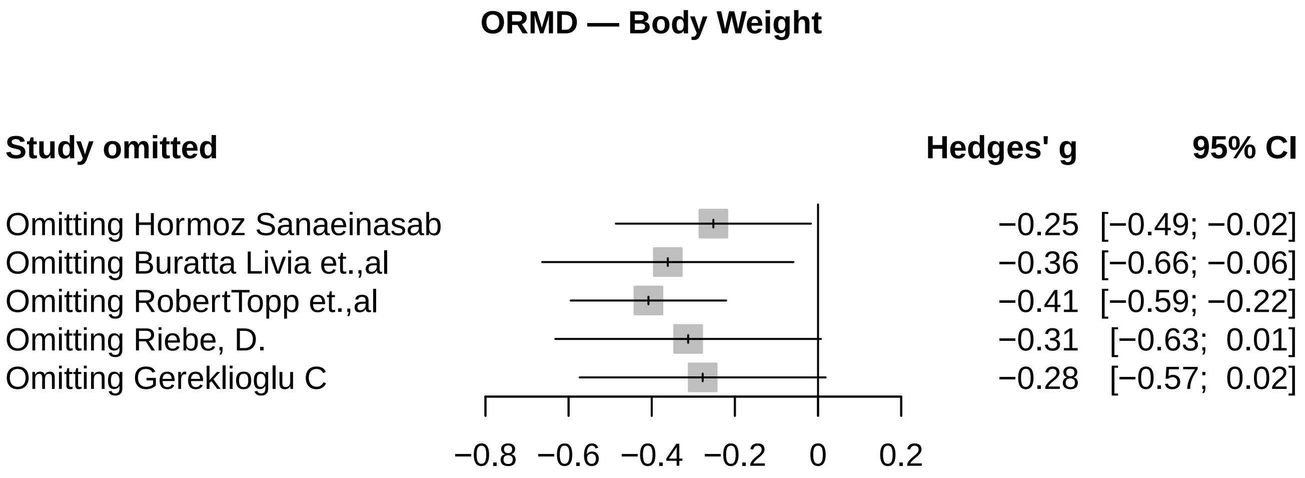
ORMD — Body Weight
Study omitted
Hedges' g
95% CI
Omitting Hormoz Sanaeinasab −0.25 [−0.49; −0.02]
Omitting Buratta Livia et.,al −0.36 [−0.66; −0.06]
Omitting RobertTopp et.,al −0.41 [−0.59; −0.22]
Omitting Riebe, D. −0.31 [−0.63; 0.01]
Omitting Gereklioglu C −0.28 [−0.57; 0.02]
−0.8 −0.6 −0.4 −0.2 0 0.2
