# Supplementary material for: Effects of transtheoretical model-based interventions on body mass index and other health outcomes in overweight or obese populations: a systematic review and meta-analysis
Source: Front Public Health. 2026 Jun 22;14:1832812. doi: 10.3389/fpubh.2026.1832812 (PMC13335073; doi:10.3389/fpubh.2026.1832812)

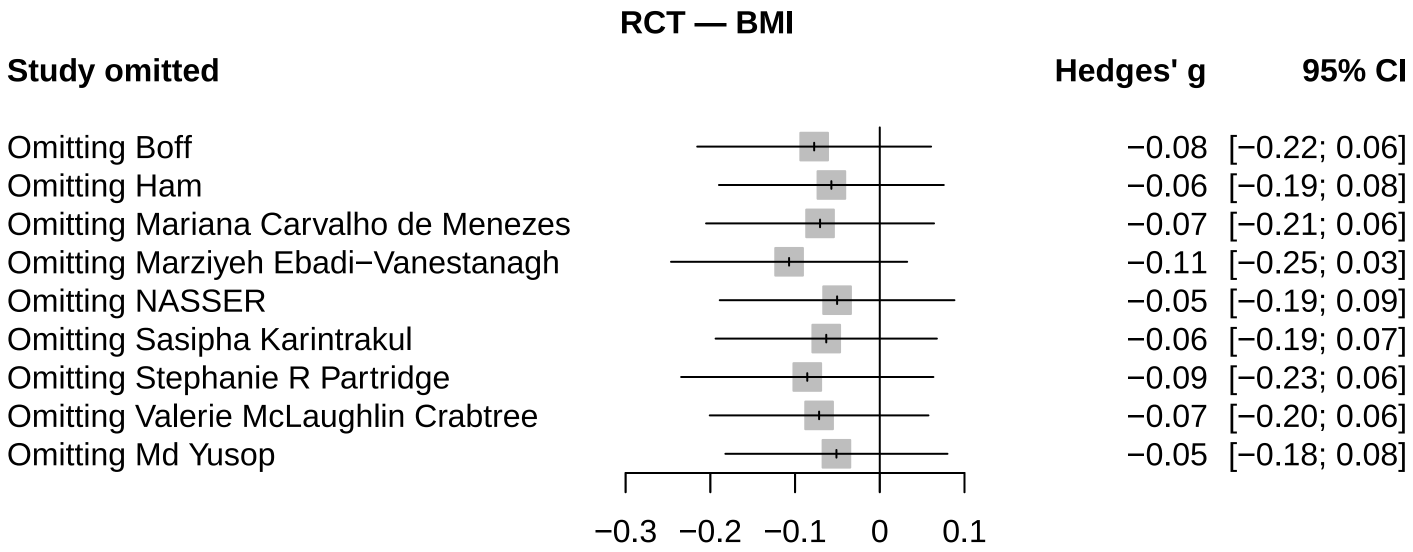
RCT — BMI
Study omitted
Hedges' g
95% CI
Omitting Boff
−0.08 [−0.22; 0.06]
Omitting Ham
−0.06 [−0.19; 0.08]
Omitting Mariana Carvalho de Menezes
−0.07 [−0.21; 0.06]
Omitting Marziyeh Ebadi−Vanestanagh
−0.11 [−0.25; 0.03]
Omitting NASSER
−0.05 [−0.19; 0.09]
Omitting Sasipha Karintrakul
−0.06 [−0.19; 0.07]
Omitting Stephanie R Partridge
−0.09 [−0.23; 0.06]
Omitting Valerie McLaughlin Crabtree
−0.07 [−0.20; 0.06]
Omitting Md Yusop
−0.05 [−0.18; 0.08]
−0.3
−0.2
−0.1
0
0.1

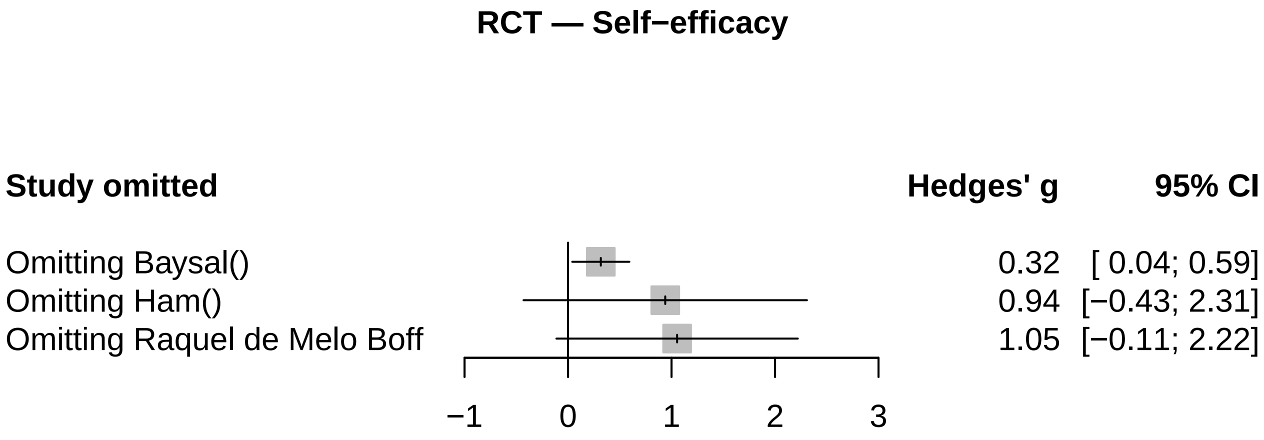
RCT — Self−efficacy
Study omitted
Hedges' g
95% CI
Omitting Baysal()
0.32
[0.04; 0.59]
Omitting Ham()
0.94
[−0.43; 2.31]
Omitting Raquel de Melo Boff
1.05
[−0.11; 2.22]
−1
0
1
2
3

RCT — Body Weight

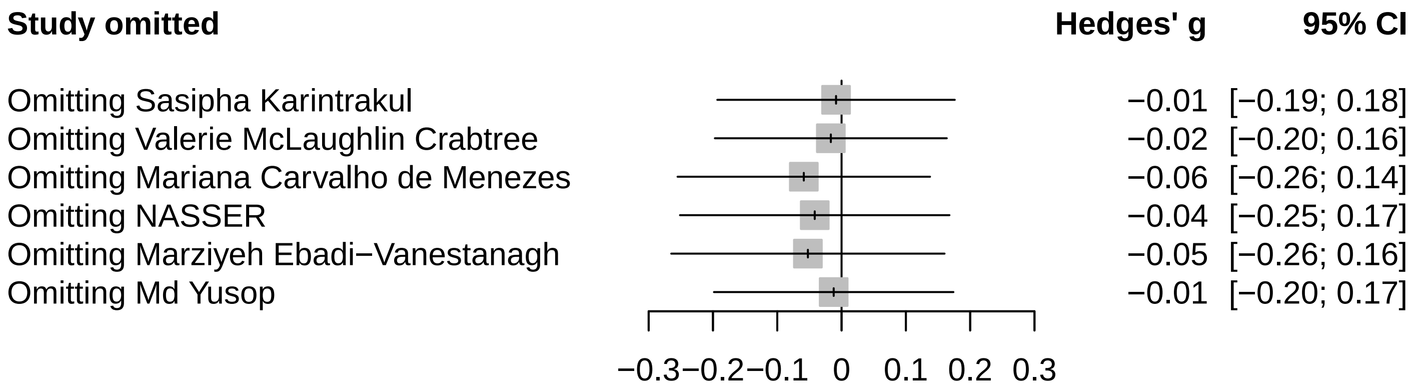

RCT — Waist Circumference

Study omitted	Hedges' g	95% CI
Omitting Sasipha Karintrakul	−0.15	[−0.35; 0.05]
Omitting Mariana Carvalho de Menezes	−0.01	[−0.33; 0.30]
Omitting NASSER	−0.04	[−0.42; 0.33]
Omitting Marziyeh Ebadi−Vanestanagh	0.00	[−0.31; 0.31]

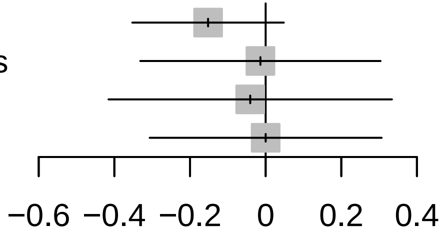

Supplement: Supplementary file 2 [file Data_Sheet_2.pdf]
